# Effect of mild hypothermia on lung injury after cardiac arrest in swine based on lung ultrasound

**DOI:** 10.1186/s12890-019-0958-8

**Published:** 2019-11-05

**Authors:** Chunshuang Wu, Jiefeng Xu, Xiaohong Jin, Qijiang Chen, Zilong Li, Mao Zhang

**Affiliations:** 1 Department of Emergency Medicine, Second Affiliated Hospital, Zhejiang University School of Medicine, Institute of Emergency Medicine, Zhejiang University, Hangzhou, China; 2 Research fellow, from Department of Emergency Medicine, Yuyao People’s Hospital, Ningbo, China; 3 Research fellow, from Department of Emergency Medicine, Wenling People’s Hospital, Taizhou, China; 4 Research fellow, from Department of Emergency Medicine, Ninghai People’s Hospital, Ningbo, China

**Keywords:** Cardiac arrest, Lung injury, Lung ultrasound, Post-cardiac arrest syndrome, Therapeutic hypothermia

## Abstract

**Background:**

Lung injury is common in post-cardiac arrest syndrome, and is associated with increased morbidity and mortality. The aim of this study was to evaluate the effect of mild hypothermia on lung injury after cardiac arrest in swine based on lung ultrasound.

**Methods:**

Twenty-three male domestic swine weighing 36 ± 2 kg were randomly assigned to three groups: therapeutic hypothermia (TH, *n* = 9), normothermia (NT, *n* = 9), and sham control (control, *n* = 5) groups. Sham animals only underwent surgical preparation. The animal model was established with 8 min of ventricular fibrillation followed by 5 min of cardiopulmonary resuscitation. Therapeutic hypothermia was induced and maintained until 24 h post-resuscitation in the TH group by surface blanket cooling, followed by rewarming at a rate of 1 °C/h for 5 h. The extravascular lung water index (ELWI), pulmonary vascular permeability index (PVPI), PO_2_/FiO_2_, and lung ultrasound score (LUS) were measured at baseline and at 1, 3, 6, 12, 24, and 30 h after resuscitation. After euthanizing the swine, their lung tissues were quickly obtained to evaluate inflammation.

**Results:**

After resuscitation, ELWI and PVPI in the NT group were higher, and PO_2_/FiO_2_ was lower, than in the sham group. However, those measures were significantly better in the TH group than the NT group. The LUS was higher in the NT group than in the sham group at 1, 3, 6, 12, 24, and 30 h after resuscitation. The LUS was significantly better in the TH group compared to the NT group. The lung tissue biopsy revealed that lung injury was more severe in the NT group than in the TH group. Increases in LUS were highly correlated with increases in ELWI (r = 0.613; *p* < 0.001) and PVPI (r = 0.683; *p* < 0.001), and decreases in PO_2_/FiO_2_ (r = − 0.468; *p* < 0.001).

**Conclusions:**

Mild hypothermia protected against post-resuscitation lung injury in a swine model of cardiac arrest. Lung ultrasound was useful to dynamically evaluate the role of TH in lung protection.

## Background

The significant morbidity and mortality of resuscitated patients are largely due to post-cardiac arrest syndrome, which is caused by whole-body ischemia and reperfusion [1]. Therapeutic hypothermia (TH) has subsequently entered the guidelines as standard therapy for this syndrome [2, 3]. A series of studies have confirmed that TH provides protection from cardiac and neurological damage after resuscitation. Lung injury is another major component of post-cardiac arrest syndrome [4]. In non-cardiac arrest models, hypothermia attenuates ischemia-reperfusion lung injury, ventilator-induced lung injury, toxin-induced lung injury, and pneumonia [5–7]. The mechanism of lung injury after resuscitation is more complex, and there have been relatively few studies on the effect of TH on lung injury.

In general, chest X-ray and computed tomography (CT) are commonly used to monitor the progression of lung injury [8, 9], but are impractical for real-time measurements, particularly in post-resuscitation patients. Although invasive measurements using conductance catheters (PiCCO device) allow for continuous, reliable, precise, and global imaging of cardiopulmonary conditions in resuscitated patients [9], they lead to a number of complications. Thus, techniques that allow for rapid and noninvasive bedside assessments of lung injury are needed for post-resuscitation patients. A lung ultrasound assessment can be easily and quickly performed at the bedside. It captures both artifacts and effusion and consolidation, which together provide information that is important for diagnosing and monitoring acute respiratory disorders [10], guiding the positive end-expiratory pressure setting, and detecting early respiratory complications in mechanically ventilated patients. Lung ultrasound can also aid the weaning process [11]. Some studies have suggested that lung ultrasound could reduce the need for standard chest radiography and CT in critically ill patients [12].

Ease of use, rapidity, repeatability, reliability, efficiency, and safety of lung ultrasound make it a principal modality for bedside assessments of the progression of lung injury in critically ill patients [13]. In cardiac arrest patients, lung ultrasound not only noinvasively provides dynamic information about respiratory disease but also does not discontinue TH therapy, or require high-risk transport. However, there have been relatively few studies on the value of lung ultrasound in post-resuscitation patients with mild hypothermia.

In this study, we assessed the protective role of mild hypothermia against lung injury after resuscitation, and whether lung ultrasound could dynamically evaluate the role of TH in lung protection.

## Methods

All animals received humane care in compliance with the “Principles of Laboratory Animal Care” of the National Society for Medical Research, and the Guide for the Care and Use of Laboratory Animals of the Institute of Laboratory Animal Resources. Healthy male white domestic swine (age, 4–6 months; weight, 36 ± 2 kg) were supplied by Shanghai Jiagan Biotechnology Inc. (Shanghai, China). The research animals were fed under conditions of standard atmospheric pressure, a 12/12-h light/dark cycle, room temperature (20–25 °C), and 60–80% humidity; cages were closed, libitum access, and there was a regular schedule of feeding, cleaning, and disinfection. This experimental study protocol was approved by the Animal Care and Use Committee of the Medical School of Zhejiang University. The data, analytical methods, and materials used in this study are available to other researchers seeking to reproduce the results or replicate the procedures, upon reasonable request to the corresponding author.

### Animal preparation

A similar anesthesia protocol and animal preparation procedure were detailed in our previous study [14]. Twenty-three male domestic pigs were fasted but had free access to water 12 h before the experiment. Ketamine (20 mg/kg) was intramuscularly injected to initiate anesthesia and sodium pentobarbital (30 mg/kg) was injected through an ear vein for complete anesthesia. Anesthesia was maintained by administering sodium pentobarbital (8 mg/kg/h) and fentanyl (2 μg/kg/h). Tracheal intubation and mechanical ventilation (SynoVent E5; Mindray, Shenzhen, China) were performed (volume-controlled mode; tidal volume of 12 mL/kg, peak flow of 40 L/min, and fraction of inspired oxygen [FiO_2_] of 0.21) to maintain end tidal carbon dioxide (ETCO_2_) between 35 and 40 mmHg (PMSH-300; SunLife Science Inc., Shanghai, China). Lead II electrocardiographic monitoring was continuously performed.

A continuous hemodynamic monitoring monitor PICCO monitor (BeneView T6; Mindray, Shenzhen, China) was applied. A polyethylene pressure transducer catheter was inserted into the right femoral artery, and another was placed in the left external jugular vein. Both catheters were connected to the monitor for continuous measurement of the extravascular lung water index (ELWI), the pulmonary vascular permeability index (PVPI), arterial pressure, and blood temperature. Another pressure transducer catheter was inserted into the right atrium, from the right femoral vein, to measure right atrial pressure. A final catheter (EP Technologies Inc., Mountainview, CA, USA) was inserted into the right external jugular vein, and directly into the right ventricle to induce ventricular fibrillation (VF). Blood temperature was maintained at 38.0 ± 0.5 °C using a cooling/warm mattress during the baseline measurements.

### Experimental procedure

#### Animal randomization

Baseline measurements were obtained 15 min prior to inducing VF. The animals were randomized into three groups using the sealed envelope method: 1) therapeutic hypothermia (TH) group, 2) normothermia (NT) group, and 3) sham control (control) group.

#### Induction of VF and cardiopulmonary resuscitation

**Fig. 1:**
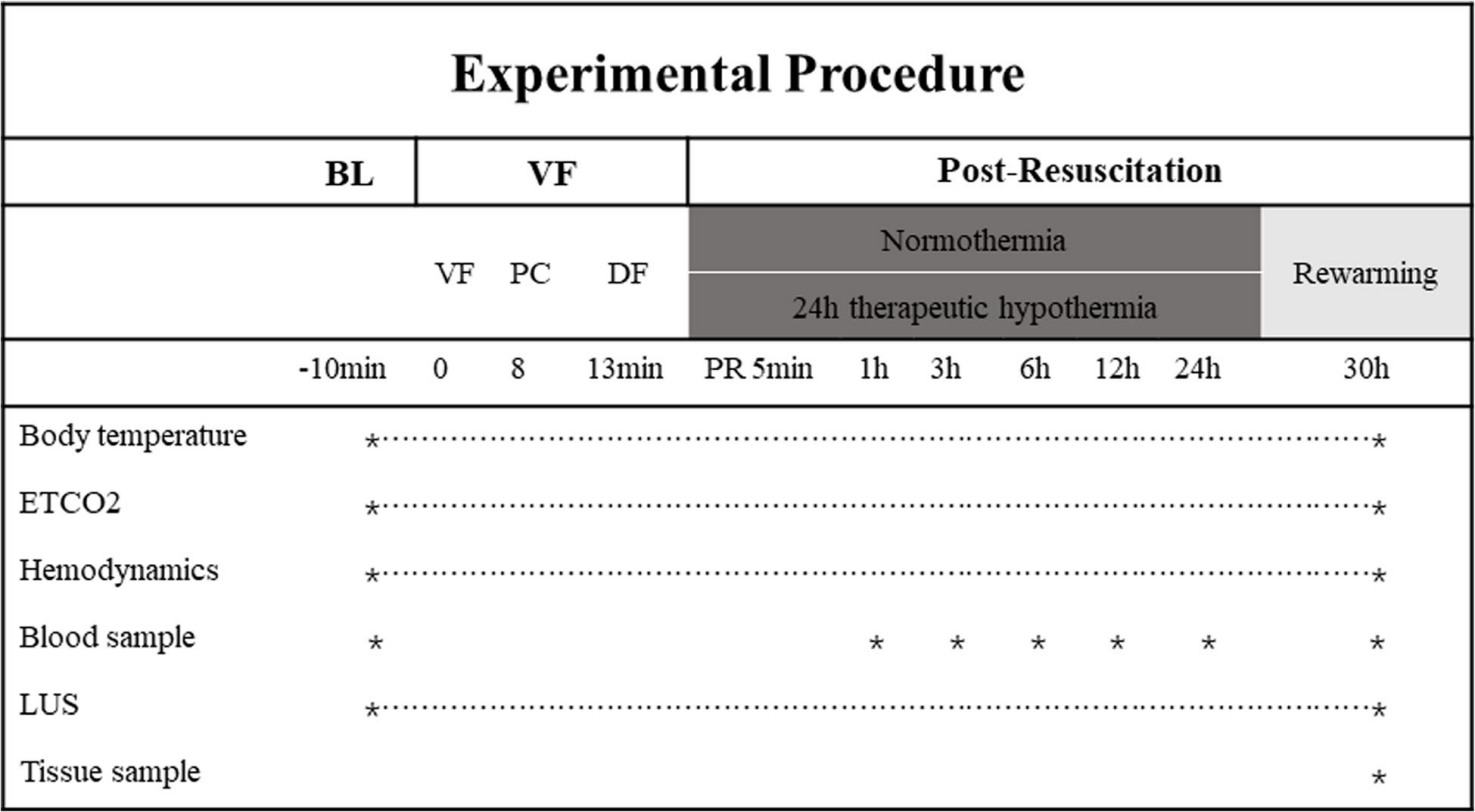
Experimental pipeline and procedure. BL indicates baseline; DF, defibrillation; PC, precordial compression; PR, post resuscitation; VF, ventricular fibrillation; ETCO2, End-Tidal Carbon Dioxide; LUS, lung ultrasound score

VF was induced by 1 mA of alternating current. Ventilation was stopped during VF. After 8 min of non-interventional VF, and before cardiopulmonary resuscitation (CPR), the pacing catheter was pulled out to avoid heart injury during chest compressions. Manual CPR was performed with a 30:2 ratio of compression to ventilation. Compression quality was continuously monitored by an R Series monitor/defibrillator (ZOLL Medical Corp., Chelmsford, MA, USA) to guarantee effective compressions (depth of 50–60 mm and rate of 100–120 per min). Ventilation was provided using a simple bag respirator and room air. Epinephrine (20 μg /kg) was administered after 2.5 min. After 5 min of CPR, defibrillation was attempted with a single 150 J biphasic waveform electrical shock delivered between the conventional right infraclavicular electrode and the apical electrode and monitored with the R Series monitor/defibrillator. Return of spontaneous circulation (ROSC) was defined as an organized rhythm with a mean arterial pressure > 50 mmHg persisting for > 5 min. If ROSC was not achieved, chest compressions and ventilation were immediately performed for 2 min prior to another defibrillation attempt. The CPR was repeated until successful resuscitation or for a total of 15 min. Additional doses of epinephrine were given at intervals of 3 min after the first bolus injection. If recurrent VF occurred after resuscitation, an additional 150 J electrical shock was attempted. TH was implemented after 5 min of resuscitation using a cooling blanket and ice packs to reach a temperature of 33 °C until 24 h after resuscitation, followed by a rewarming rate of 1 °C/h over 5 h in the TH group. In the NT and control groups, the temperature was maintained at 37–38 °C during the entire 30 h period. The experimental pipeline is summarized in Fig. 1.

### Measurements

The ventilator parameters remained unchanged throughout the experiment in the control group, and were unchanged before and after resuscitation in the NT and TH groups to avoid an effect on pulmonary injury after resuscitation. So they were never changed in any group. Electrocardiogram, mean artery pressure, blood temperature, and pulse oxygen saturation were continuously recorded. Coronary perfusion pressure was calculated as the difference between aortic pressure and right atrial pressure measured at the end of each minute of compression. ETCO_2_ was recorded continuously by a handheld ETCO_2_/SPO_2_ monitor.

PiCCO monitoring of ELWI and PVPI were evaluated at baseline and 1, 3, 6, 12, 24, and 30 h after resuscitation. After injecting 15 mL of 0 °C 0.9% saline bolus into the central vein of each animal, arterial temperature was determined using a temperature sensor placed in the femoral artery catheter, and ELWI and PVPI were calculated. The final ELWI and PVPI were the average of three consecutive injections. Aortic blood pH, partial pressure of carbon dioxide (PCO_2_), partial pressure of oxygen (PO_2_), and hemoglobin and lactate concentrations were measured at specific time points using 1.5 mL arterial blood samples with a blood gas/electrolyte analyzer (Model 5700; Instrumentation Laboratory, Lexington, MA, USA).

#### Lung ultrasound score (LUS)

**Fig. 2:**
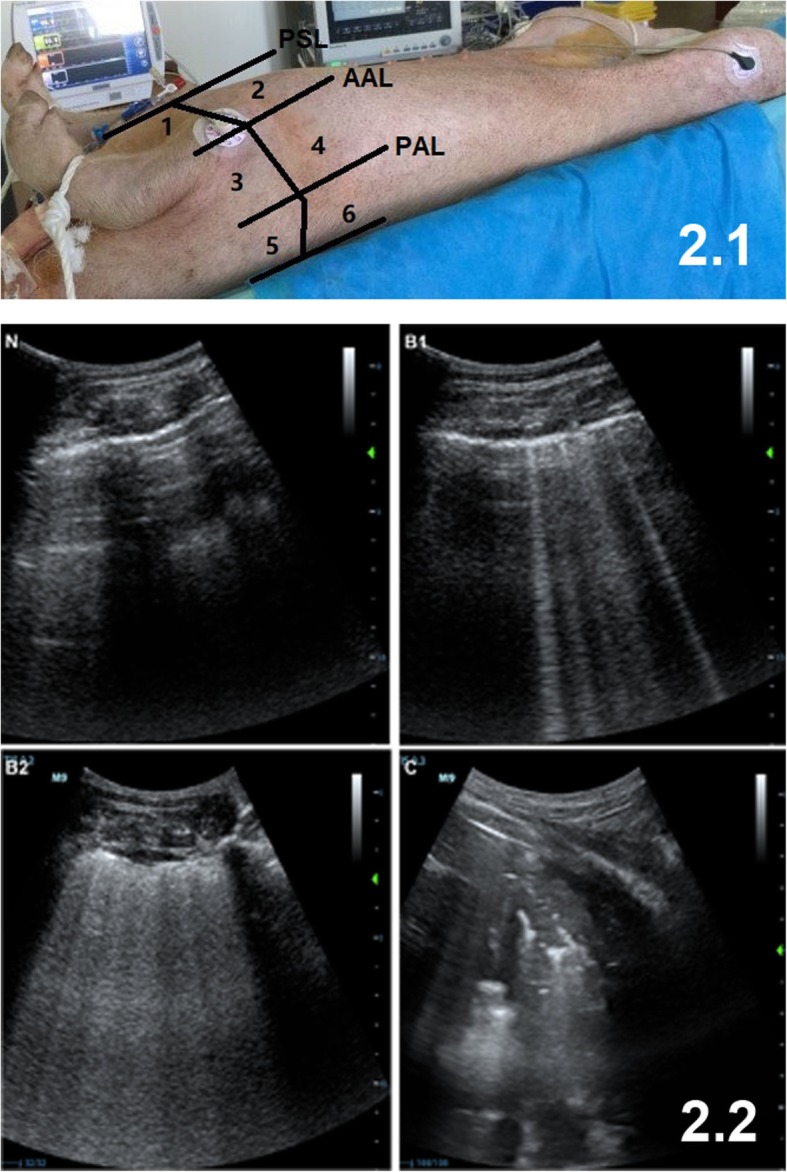
Lung ultrasound scanning. 2.1. Twelve regions of the left and right chest wall in lung ultrasound scanning. PSL, parasternal line; AAL, anterior axillary line; PAL, posterior axillary line. The upper and lower portions are bounded by the midpoint of the sternum; 2.2. Lung ultrasound pattern. N normal (zero); B1 moderate (one point); B2 severe (two points); and C lung consolidation (three points)

LUS was determined using a convex probe (M9; Mindray, Shenzhen, China) by a trained researcher (CSW) at baseline and at 1, 3, 6, 12, 24, and 30 h, as descried previously [15]. The same investigator undertook all LUS values at each time point. The LUS for the whole lung was obtained during a 10-min period. Ultrasound scanning of 12 regions of the left and right chest wall was performed (Fig. 2): the upper and lower parts were bounded by the midpoint of the sternum. The anterior, lateral, and posterior regions were divided by the anterior and posterior axillary lines.

Four ultrasound aeration patterns were defined [11, 16]: 1) normal aeration (N): presence of lung sliding with A lines or fewer than two isolated B lines; 2) moderate loss of lung aeration: multiple, well-defined B lines (B1 lines); 3) severe loss of lung aeration: multiple coalescent B lines (B2 lines); and 4) lung consolidation (C), the presence of a tissue pattern characterized by dynamic air bronchograms. Points were allocated to any region of interest, according to the worst ultrasound pattern observed (Fig. 2): N = 0, B1 lines = 1, B2 lines = 2, C = 3. A LUS value ranging between 0 and 36 was calculated by summing the points. All ultrasound images were stored for retrospective analyses.

#### Histological analysis

The animals were euthanized with an intravenous injection of 150 mg/kg pentobarbital. Specimens were taken from lower left lung lobes, rinsed in saline, and immersed in 10% formalin fixative for 24 h. After washing with water, they were sliced to a 1-cm thickness. The lung tissues were dehydrated through a graded alcohol series and then embedded in paraffin at 60 °C for the pathological light microscopic examination. Sections (4.5 μm thick) were stained with hematoxylin and eosin. The degree of lung injury was assessed qualitatively according to the fibrin effusion in the alveoli, neutrophil infiltration and effusion in the alveoli, interalveolar septal thickening, and microthrombus formation in the capillaries [17]. The specimens were collected, treated and analyzed by a laboratory technician who was not directly involved in the experimental process.

### Statistical analyses

The statistical analysis was performed using IBM SPSS software (version 19.0; IBM Corp., Armonk, NY, USA). Continuous variables are presented as the mean ± standard deviation for normally distributed data. Changes in ELWI, PVPI, PO_2_/FiO_2_, and LUS over time were compared using a repeated-measures analysis. The Bonferroni test was used to compare two groups. Continuous variables were compared (pairwise) among the three groups at the same time points with Student’s *t*-test. The correlations between the LUS and traditional indices (ELWI, PVPI, or PO_2_/FiO_2_) were determined using Pearson’s correlation coefficient analyses. *P*-values < 0.05 were considered significant.

## Results

Twenty-three experiments were performed. No significant differences were observed in the baseline hemodynamics or body temperature among the three groups (Table 1). Coronary perfusion pressure, duration of CPR, number of defibrillations, and total dose of epinephrine administered were not different between the TH and NT groups during CPR (Additional file 1 Table. S1). One pig each in the TH and NT groups did not achieve ROSC. Another pig in the NT group survived < 12 h after ROSC. Thus, 20/23 pigs were successfully resuscitated after cardiac arrest and were then observed for up to 30 h.

**Table 1:** Baseline characteristics

Variables	TH group (*n* = 9)	NT group (*n* = 9)	Control group(*n* = 5)	P
Body weight, kg	36.3 ± 3.1	36.9 ± 2.7	36.2 ± 3.0	0.901
Heart rate, beats/min	108.6 ± 11.0	105.4 ± 13.4	104.4 ± 5.0	0.762
Mean aortic pressure, mmHg	113.6 ± 12.0	121.4 ± 12.4	119.8 ± 7.8	0.395
End-tidal CO2, mmHg	39.4 ± 3.3	40.1 ± 2.9	39.6 ± 1.7	0.872
Core temperature, °C	37.9 ± 0.3	38.0 ± 0.3	37.9 ± 0.4	0.891
ROSC	8/9	7/9	5/5	0.172
Duration of CPR, min	5 ± 0	5.6 ± 1	_	0.158
Number of shocks to ROSC	1.75 ± 1.4	3.29 ± 2.5	_	0.084
Epinephrine dosage, mg	71.5 ± 71.5	1025.7 ± 395.6	_	0.345
Prevalence of recurrent VF	0.8 ± 1.4	1.7 ± 2.4	_	0.762

*TH*, Therapeutic hypothermia; *NT*, Normothermia; *ROSC*, Return of sponstaneous circulation; *CPR*, Cardiopulmonary resuscitation; *VF*, Ventricular fibrillation

Data are presented as mean ± SD

**Fig. 3:**
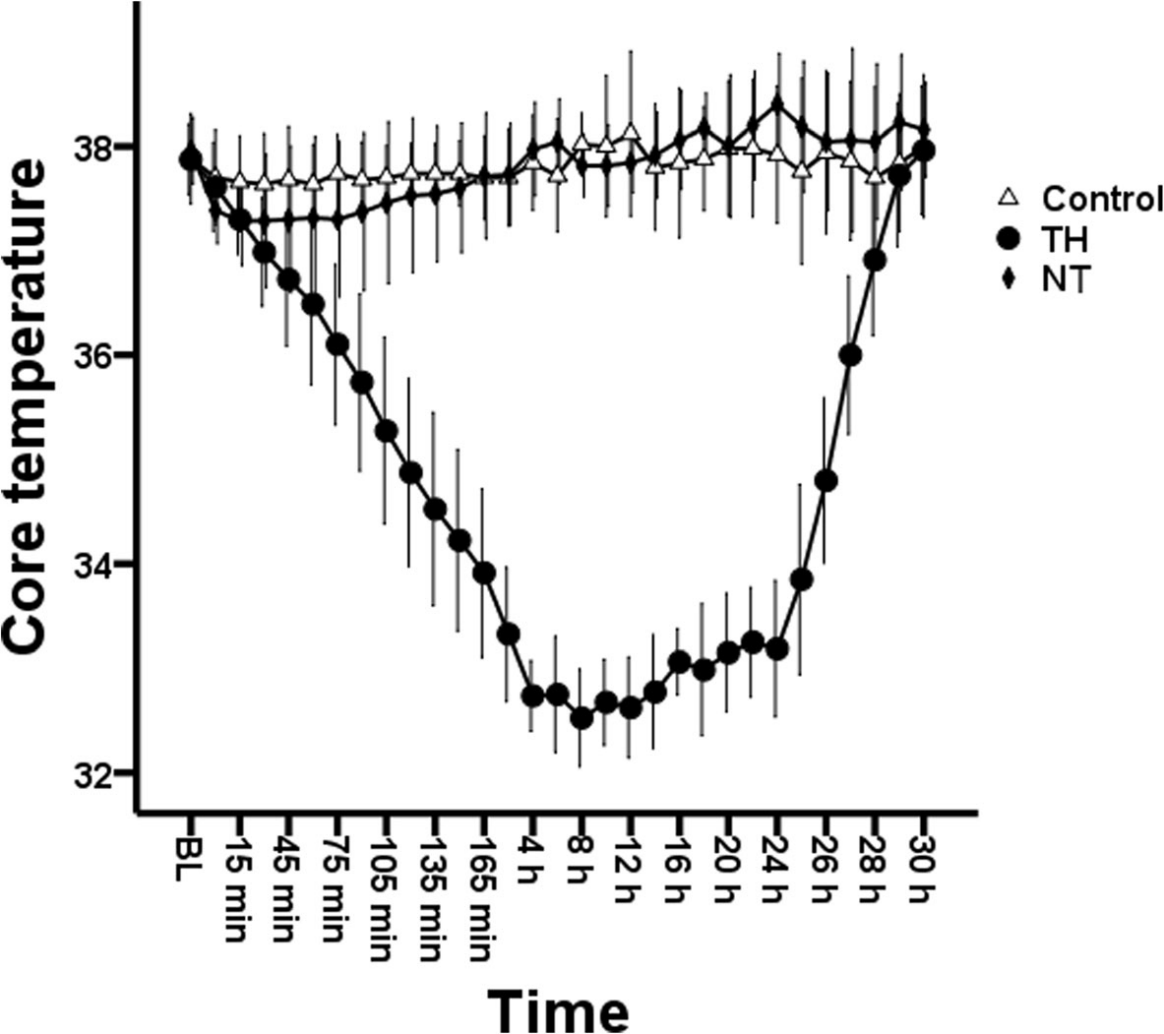
Core temperature after resuscitation. TH, therapeutic hypothermia; NT, normothermia; BL, baseline

Body temperature decreased rapidly from 37.9 ± 0.3 to 34.9 ± 0.9 °C within 2 h after resuscitation in the TH group. Thereafter, a temperature of 33 °C was maintained until 24 h after ROSC, followed by a rewarming rate of 1 °C/h for 5 h (Fig. 3). Blood temperature was maintained at 37–38 °C throughout the study in the control and NT groups.

After the 30-h observation, no differences in the indices of interest from baseline were seen in the sham animals. The indices in TH group were more stable than those in the NM group. PO_2_/FiO_2_ decreased overall from 438 ± 77 to 289 ± 82 (*p* < 0.05) in the NM group, and from 475 ± 37 to 380 ± 33 (*p* < 0.05) in the TH group. ELWI increased overall from 9.71 ± 1.25 to 18.43 ± 3.60(*p* < 0.05) in the NM group, and from 9.50 ± 1.51 to 13.88 ± 1.96 (*p* < 0.05) in the TH group; PVPI changed from 2.41 ± 0.59 to 5.69 ± 0.71 and 2.31 ± 0.42 to 4.23 ± 0.73, respectively (*p* < 0.05). In the TH group, the ELWI, and PVPI were significantly higher than in the NM group after both 24 and 30 h; PO_2_/FiO_2_ in the TH group was significantly lower than in the NM group at 12, 24, and 30 h.

Each LUS assessment was performed in less than 10 min, with full feasibility of 100%. The mean baseline LUS values in the control, TH, and NT groups were 1.00, 1.63, and 1.25, respectively (*p* = 0.616). After ROSC, the score increased over time and more markedly 1 h later with a mean of 8.88 (*p* < 0.001) and 10.38 (*p* < 0.001) in the TH and NT groups, respectively. LUS then remained stable. The LUS value of the NT group was significantly higher at 3, 6, and 12 h than that in the TH group (*p* < 0.05) (Table 2).

**Table 2:** Dynamic changes of LUS, PO2/FiO2, ELWI, PVPI in three groups after CPR

		Baseline	Post resuscitation						*F*
			1 h	3 h	6 h	12 h	24 h	30 h	
PO2/FiO2									6.195*
	Control	440.0 ± 56.5	449.6 ± 38.4	443.6 ± 48.0	429.4 ± 39.7	426.6 ± 31.8	433.2 ± 32.7	439.8 ± 19.4	
	NT	438.0 ± 77.0	342.0 ± 44.4^*a*^	343.7 ± 58.9^*a*^	322.4 ± 41.1^*a*^	332.4 ± 75.2^*a*^	278.7 ± 94.4^*a*^	289.3 ± 82.2^*a*^	
	TH	463.0 ± 47.9	367.4 ± 45.2	385.9 ± 74.4	414.1 ± 51.5	410.8 ± 42.4^*b*^	400.5 ± 37.5^*b*^	378.6 ± 31.1^*b*^	
*F*	12.962^#^								2.596^†^
ELWI									25.348*
	Control	9.2 ± 0.8	10.4 ± 1.1	10.8 ± 1.3	9.8 ± 1.1	10.2 ± 1.3	10.2 ± 1.6	10.0 ± 1.4	
	NT	9.7 ± 1.3	17.1 ± 3.6^*a*^	16.7 ± 4.1^*a*^	18.3 ± 4.3^*a*^	20.4 ± 4.7^*a*^	20.9 ± 3.0^*a*^	18.4 ± 3.6^*a*^	
	TH	9.5 ± 1.5	13.6 ± 2.4	17.3 ± 3.3^*a*^	17.4 ± 3.1^*a*^	17.4 ± 3.3^*a*^	16.0 ± 3.6^*ab*^	13.9 ± 2.0^*b*^	
*F*	13.562^#^								5.614^†^
PVPI									27.906*
	Control	2.4 ± 0.3	2.5 ± 0.7	2.7 ± 0.5	2.7 ± 0.5	2.5 ± 0.9	2.5 ± 0.3	2.6 ± 0.6	
	NT	2.4 ± 0.6	4.5 ± 0.7^*a*^	5.2 ± 1.1^*a*^	5.9 ± 1.2^*a*^	6.1 ± 0.8^*a*^	6.2 ± 1.1^*a*^	5.7 ± 0.7^*a*^	
	TH	2.3 ± 0.4	4.1 ± 0.8^*a*^	4.8 ± 0.8^*a*^	4.7 ± 1.0^*a*^	5.0 ± 1.1^*a*^	4.6 ± 1.0^*ab*^	4.2 ± 0.7^*ab*^	
*F*	27.249^#^								2.657^†^
LUS									27.115*
	Control	1.0 ± 1.0	3.6 ± 1.3	3.6 ± 1.3	3.2 ± 1.6	3.8 ± 0.8	3.0 ± 1.4	4.4 ± 2.1	
	NT	2.3 ± 0.8	12.3 ± 2.2^*a*^	13.3 ± 3.5^*a*^	14.0 ± 3.5^*a*^	15.4 ± 5.0^*a*^	12.4 ± 3.1^*a*^	13.9 ± 6.4^*a*^	
	TH	0.9 ± 1.0	7.9 ± 4.1	7.5 ± 2.7^*b*^	8.8 ± 1.5^*ab*^	8.6 ± 2.1^*b*^	8.8 ± 4.0^*a*^	8.3 ± 3.2	
*F*	23.359^#^								12.000^†^

Values are presented as mean ± SD

^*a*^*p* < 0.05, versus the control group

^*b*^*p* < 0.05, versus the NT group

*p < 0.05, with group effect

^#^*p* < 0.05, with time effect

^†^*p* < 0.05, with group & time interaction

*ELWI*, Extravascular lung water index; *PVPI*, Pulmonary vascular permeability index; *LUS*, Lung ultrasound score; *TH*, Therapeutic hypothermia; *NT*, Normothermia; *BL*, Baseline

**Fig. 4:**
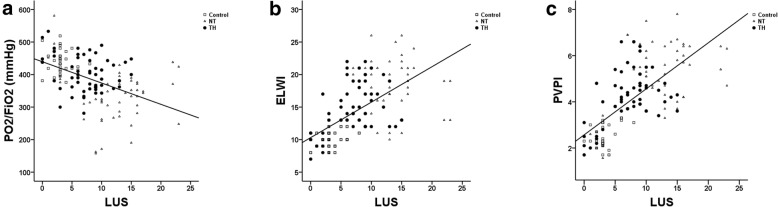
**a** Correlation between the LUS and the PO2/FiO2. **b** Correlation between the LUS and the PVPI. **c** Correlation between the LUS and the ELWI

Significant positive linear correlations were found between LUS and ELWI (r = 0.613; *p* < 0.001), and PVPI (r = 0.683; *p* <0.001). A significant negative linear correlation was observed between the LUS and PO_2_/FiO_2_ (r = − 0.468; *p* <0.001) (Fig. 4).

**Fig. 5:**
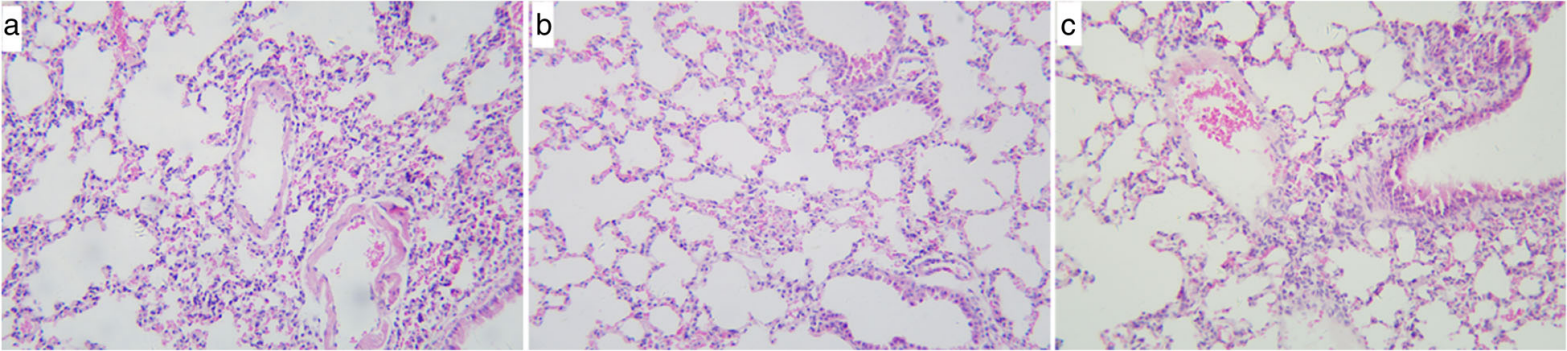
Histopathology (hematoxylin and eosin staining [H&E] 10 × 20). **a** Sham group. **b** TH group. **c** NT group

Very significant hemorrhagic edema with inflammatory cell infiltration were detected in both the NT and TH groups at 30 h post-resuscitation, compared to the sham group. The lung injury was more severe in the NT group than in the TH group (Fig. 5).

## Discussion

ELWI, PVPI, and PO_2_/FiO_2_ significantly changed in the TH and NT groups compared to the control group after resuscitation in a porcine model of VF-induced cardiac arrest. LUS provided reliable information on the pulmonary conditions, varying tendency of lung injury, and lung protection by hypothermia after cardiac arrest.

The clinical data on cardiac arrest were limited and varied, which made comparisons difficult, and the results were equivocal. Animal models of cardiac arrest allow for well-controlled conditions, where equivalent measurements cannot be obtained in humans; thus, animal models provide a unique opportunity to explore new monitoring techniques and therapies. Thus, we chose large animals for the preliminary exploration in this study. Pigs are among the most commonly used animals for cardiac arrest models; they are highly comparable with humans with regard to size, physiology, and anatomy, and the pig heart and cardiovascular physiology share many similarities with those of humans [18]. Furthermore, our earlier studies showed that our pig model is feasible to explore the monitoring of, and protection against, multiple organ injuries (e.g., lung injuries) after resuscitation [14, 19, 20]. Therefore, this experimental study using pigs explored whether mild hypothermia had a protective effect against post-resuscitation lung injury, and lung ultrasound was used to dynamically evaluate the role of TH in lung protection.

ELWI, PVPI, and PO_2_/FiO_2_ are traditional markers of the severity of lung injury. In this study, ELWI, PVPI, and PO_2_/FiO_2_ changed significantly in the TH and NT groups compared to the control group after resuscitation, indicating the presence of lung injury after cardiac arrest in both of the former groups. Previous studies have shown that lung injury is common after resuscitation [21, 22]. The mechanism of lung injury after cardiac arrest is unclear for a variety of reasons; first, pulmonary hemorrhage and edema may be initiated by the alveolar capillary membrane rupture caused by chest compressions; second, oxidative stress induced by the generation of intracellular free radicals may aggravate the damage to membrane function and lead to ischemia-reperfusion injury; third, aspirated gastric juices and oropharyngeal secretions during chest compressions can also cause lung damage [23, 24].

TH is an important component of post-cardiac arrest treatment [3]. The benefits of TH with respect to neurological and cardiac outcomes have been documented extensively, but knowledge regarding its effects on post-resuscitation lung injury is limited [25, 26]. A previous study by Su indicated that hypothermia reduces the release of pro-inflammatory mediators, inhibits ATPase activity in alveolar membranes, and protects lung tissue from hypoperfusion [27]. In our study, the traditional lung injury indices improved significantly in the TH group compared to those in the NT group, thereby demonstrating the role of TH in lung protection.

CT or magnetic resonance imaging is the gold standard imaging modality for lung injury, but is not appropriate for convenient and repeated assessments. PiCCO technology has become widely used in clinics, and ELWI and PVPI are used to estimate lung water and permeability. However, the device is expensive, invasive, may lead to complications, and could provide no clinical benefit [9]. Therefore, techniques that allow for rapid and noninvasive bedside assessments are needed. In recent years, lung ultrasound has been increasingly used in daily practice in the intensive care unit [28, 29]. This method allows for patterns of progressive loss of aeration to be distinguished [30], and has shown its usefulness to monitor ventilated patients [31, 32]. Agricola et al. [33] and Zhao et al. [31] reported a strong correlation between lung ultrasound and extravascular lung water (EVLW) measured using trans-pulmonary thermodilution and indicated that the early LUS is a good prognostic index in patients with acute respiratory distress syndrome. Jambrik et al. [34] detected a linear correlation between the echocardiographic comet score and the radiological extravascular lung water score in hospitalized patients. Picano et al. [35] suggested that the number of B lines can be used to generate a quantitative or semi-quantitative score to evaluate EVLW for pulmonary congestion. Ultrasound, as a viable bedside technique, is being increasingly used to assess lung aeration, and changes [36]. A correlation between LUS and quantitative CT scan or EVLW data has been reported in patients suffering from acute respiratory failure, and in the management of circulatory failure [37, 38]. However, the mechanisms of lung injury after resuscitation are complex, and there is little evidence that ultrasound can be used to evaluate post-resuscitation lung injury. In this study, LUS was used to dynamically monitor pulmonary condition and the lung-protective effects of hypothermia, and was well correlated with the ELWI, PVPI, and PO_2_/FiO_2_. Lung ultrasound is a feasible bedside tool to dynamically monitor lung injury. Nevertheless, future clinical work is necessary to establish a defined method for grading lung injury severity by ultrasonography [39].

Some limitations of this study should be acknowledged. First, although all of the tests showed significant differences among the groups, the number of animals was small. Second, the rewarming rate was 1 °C/h, while the guidelines recommend a rate of 0.25–0.5 °C/h [2]. However, this did not affect the beneficial effects of TH, based on a previous study [40]. Third, CT is the gold standard imaging modality for lung injury, but was not used herein due to limited resources. Fourth, even large animal models of CPR do not fully reflect the situation in human patients. Therefore, large clinical studies are needed to confirm our results. Last, the lack of change in ventilatory parameters throughout the experiment may have led to a deterioration in lung function, which is not in line with clinical findings. Our data showed that the LUS in the TH group was better compared to the NT group, where all animals were tested under the same conditions. This study further demonstrates that lung ultrasound can be used to evaluate the protective effect of TH on lung injury.

## Conclusions

Mild hypothermia protected against post-resuscitation lung injury in a swine model of cardiac arrest. Lung ultrasound was useful for dynamically evaluating the role of TH in lung protection.

## Supplementary information

**Additional file 1: Table S1.** Coronary perfusion pressure during CPR.

## Data Availability

The data that support the findings of this study are available from the corresponding author upon reasonable request and with the permission of the institution.

## Abbreviations

CPR: Cardiopulmonary resuscitation
CT: Computed tomography
ELWI: Extravascular lung water index
ETCO_2_: End-tidal carbon dioxide
EVLW: Extravascular lung water
FiO2: Fraction of inspired oxygen
LUS: Lung ultrasound score
NT: Normothermia
PCO2: Partial pressure of carbon dioxide
PO2: Partial pressure of oxygen
PVPI: Pulmonary vascular permeability index
TH: Therapeutic hypothermia
VF: Ventricular fibrillation
