# Supplementary material for: Effect of mild hypothermia on lung injury after cardiac arrest in swine based on lung ultrasound
Source: BMC Pulm Med. 2019 Nov 5;19:198. doi: 10.1186/s12890-019-0958-8 (PMC6833209; doi:10.1186/s12890-019-0958-8)
Supplement: Supplementary file 1 — Additional file 1: Table S1. Coronary perfusion pressure during CPR. [file 12890_2019_958_MOESM1_ESM.doc]

**Table S1 Coronary perfusion pressure during CPR**

| CPP | TH group (n=8) | NT group (n=7) | P |
| --- | --- | --- | --- |
| CPP in PC 1, mmHg | 17.8±2.6 | 17.4±3.2 | 0.379 |
| CPP in PC 2, mmHg | 22.8±3.4 | 21.9±2.4 | 0.591 |
| CPP in PC 3, mmHg | 28.6±5.1 | 28.7±3.2 | 0.115 |
| CPP in PC 4, mmHg | 37.8±4.4 | 37.1±3.9 | 0.892 |
| CPP in PC 5, mmHg | 27.9±5.2 | 27.1±4.7 | 0.691 |

CPR, cardiopulmonary resuscitation; TH, therapeutic hypothermia; NT, normothermia; CPP, coronary perfusion pressure; PC*_n_*, indicates n min after precordial compression.

Data are presented as mean±SD.
